# Lack of Dopaminergic Inputs Elongates the Primary Cilia of Striatal Neurons

**DOI:** 10.1371/journal.pone.0097918

**Published:** 2014-05-15

**Authors:** Ko Miyoshi, Kyosuke Kasahara, Shinki Murakami, Mika Takeshima, Natsuko Kumamoto, Asako Sato, Ikuko Miyazaki, Shinsuke Matsuzaki, Toshikuni Sasaoka, Taiichi Katayama, Masato Asanuma

**Affiliations:** 1 Department of Child Development and Molecular Brain Science, United Graduate School of Child Development, Osaka University, Suita, Japan; 2 Molecular Research Center for Children's Mental Development, United Graduate School of Child Development, Osaka University, Suita, Japan; 3 Department of Brain Science, Okayama University Graduate School of Medicine, Dentistry and Pharmaceutical Sciences, Okayama, Japan; 4 Department of Neurobiology and Anatomy, Nagoya City University Graduate School of Medical Sciences, Nagoya, Japan; 5 Department of Laboratory Animal Science, Kitasato University School of Medicine, Sagamihara, Japan; 6 Department of Anatomy and Neuroscience, Graduate School of Medicine, Osaka University, Suita, Japan; 7 Department of Comparative and Experimental Medicine Center for Bioresource-based Researches, Brain Research Institute, Niigata University, Niigata, Japan; Florey Institute of Neuroscience and Mental Health, The University of Melbourne, Australia

## Abstract

In the rodent brain, certain G protein-coupled receptors and adenylyl cyclase type 3 are known to localize to the neuronal primary cilium, a primitive sensory organelle protruding singly from almost all neurons. A recent chemical screening study demonstrated that many compounds targeting dopamine receptors regulate the assembly of *Chlamydomonas reinhardtii* flagella, structures which are analogous to vertebrate cilia. Here we investigated the effects of dopaminergic inputs loss on the architecture of neuronal primary cilia in the rodent striatum, a brain region that receives major dopaminergic projections from the midbrain. We first analyzed the lengths of neuronal cilia in the dorsolateral striatum of hemi-parkinsonian rats with unilateral lesions of the nigrostriatal dopamine pathway. In these rats, the striatal neuronal cilia were significantly longer on the lesioned side than on the non-lesioned side. In mice, the repeated injection of reserpine, a dopamine-depleting agent, elongated neuronal cilia in the striatum. The combined administration of agonists for dopamine receptor type 2 (D2) with reserpine attenuated the elongation of striatal neuronal cilia. Repeated treatment with an antagonist of D2, but not of dopamine receptor type 1 (D1), elongated the striatal neuronal cilia. In addition, D2-null mice displayed longer neuronal cilia in the striatum compared to wild-type controls. Reserpine treatment elongated the striatal neuronal cilia in D1-null mice but not in D2-null mice. Repeated treatment with a D2 agonist suppressed the elongation of striatal neuronal cilia on the lesioned side of hemi-parkinsonian rats. These results suggest that the elongation of striatal neuronal cilia following the lack of dopaminergic inputs is attributable to the absence of dopaminergic transmission via D2 receptors. Our results provide the first evidence that the length of neuronal cilia can be modified by the lack of a neurotransmitter's input.

## Introduction

Almost all vertebrate cells possess an immotile primary cilium, which is a cellular appendage with axonemal microtubules in the center wrapped by a membrane that is continuous with the plasma membrane [1], [2]. The primary cilium protrudes singly out of a basal body and transduces sensory stimuli in the extracellular milieu to the cell body [1], [2]. Dysfunctions of cilia-related molecules result in human genetic diseases collectively referred to as ciliopathies including cystic kidney disease, retinal degeneration and Bardet-Biedl syndrome (BBS) [1], [2]. In almost all brain regions of rodents, each neuron forms a solitary primary cilium [3], yet the biological roles of neuronal primary cilia remain unclear.

In the rodent brain, adenylyl cyclase type 3 (AC3) [4], [5] and a growing number of G protein-coupled receptors (GPCRs) including serotonin receptor type 6 [6], somatostatin receptor type 3 (SSTR3) [7], [8], melanin-concentrating hormone receptor 1 (MCHR1) [9], vasoactive intestinal peptide receptor 2 [10], neuropeptide Y family receptors [11] and GPR161 [12] have been found to localize to the primary cilia of neurons. In the olfactory receptor neurons, odorant signal transduction relies on specialized cilia harboring AC3 and a large family of GPCRs [13]. Thus, it is likely that G protein/cyclic AMP (cAMP) cascades in neuronal cilia also detect and amplify extracellular chemical stimuli in the central nervous system [3], [14]–[19]; in other words, the neuronal cilium acts as an extra-synaptic neurotransmission device.

Growing evidence has been elucidating the possible biological roles of primary cilia of mature neurons *in vivo*. The disruption of cilia on neurons throughout the central nervous system or on pro-opiomelanocortin-expressing cells in the hypothalamus has led to obesity in mice, indicating that neuronal cilia function in a pathway regulating satiety responses [20]. Mice systemically deficient in SSTR3 or AC3 have displayed dysfunction in memory and learning [21], [22], whereas it is possible that the phenotype is independent of signaling within the ciliary compartment.

The biflagellate green alga *Chlamydomonas reinhardtii* has been used as a model single-celled organism for the study of flagella, structures which are analogous to vertebrate cilia. Based on analyses using algae, it is widely accepted that the construction and length regulation of flagella and cilia depend on a type of machinery called intraflagellar transport (IFT), which is an anterograde and retrograde trafficking system consisting of motor molecules and IFT particles consisting of several IFT subunits [23]. While the molecular mechanism controlling the length of cilia and flagella is not fully understood, an increasing number of factors have been identified as influencing the cilia and flagella length [24].

We and Ou et al. have demonstrated that treatment with lithium, an agent used as a mood stabilizer, elongates the primary cilia of cultured mammalian cells [25], [26] and those of neurons in the mouse striatum [25]. Sharma et al. showed that the modification of microtubule and actin cytoskeletons influences the primary cilium length via the levels of soluble tubulin in the cytosol available for primary cilia extension in cultured renal collecting duct cells and retinal pigmented epithelial cells [27]. Besschetnova et al. demonstrated that an increase in the intracellular cAMP level with consequent protein kinase A activation induces an elongation of primary cilia in cultured renal collecting duct cells and embryonic kidney epithelial cells [28].

Studies using a loss-of-function RNA interference genetic screen [29], [30] revealed that the assembly of primary cilia is regulated not only by IFT constituents but by diverse genetic factors including genes associated with neuropsychiatric disorders. Further, a recent chemical screening using an annotated library of small molecules against algae has identified compounds targeting dopamine receptors as major regulators of flagellar assembly [31]. This finding led us to investigate whether deficiency of dopaminergic input affects the architecture of primary cilia of neurons that receive dopaminergic projection. Here, we show the elongation of neuronal primary cilia in the striatum of the lesioned side in hemi-parkinsonian rats, each with a unilateral 6-hydroxydopamine (6-OHDA) lesion of the nigrostriatal dopaminergic pathway. We also examined the effect of a deficiency of dopaminergic input on the lengths of striatal neuronal cilia in mice, by pharmacological and genetic analyses.

## Materials and Methods

### Ethics Statement

All experiments were performed in compliance with the Guidelines for Animal Experiments of the Okayama University Advanced Science Research Center. The experimental protocol was approved by the Animal Care and Use Committee of Okayama University.

### Animals

Adult male Sprague-Dawley rats and adult male BALB/c mice were obtained from Charles River Japan (Yokohama, Japan). The mice with genetically inactivated dopamine receptor type 1 (D1-null) or genetically inactivated dopamine receptor type 2 (D2-null) on a C57BL/6J background were as described [32], [33].

### 6-OHDA lesions in rats

Unilateral nigrostriatal dopaminergic lesions were made by 6-OHDA injection as described, with some modification [34]. Briefly, 9-week-old rats were anesthetized and secured in a stereotaxic instrument with the tooth bar set +5 mm above the interaural line. 6-OHDA hydrobromide (Sigma-Aldrich, St. Louis, MO, USA) was injected at two sites (5 µg in 2 µL per site) in the right medial forebrain bundle on day 0 at the following coordinates: −1.0 mm anterior to the bregma (AP), +1.8 mm lateral from the midline (ML), +8.5 mm below the dura (DV), and AP −1.4 mm, ML +1.5 mm, DV +8.0 mm. The sham-operated animals received vehicle only (physiological saline containing 0.01% ascorbic acid) at the same coordinates.

To confirm the lesion induction, we subjected the rats to an apomorphine-induced rotation test on day 13. Rats that displayed asymmetric rotational behavior toward the left direction after the injection of apomorphine hydrochloride hemihydrate (0.1 mg/kg, s.c., Sigma-Aldrich) were selected for further experiments. 6-OHDA-lesioned rats were perfusion-fixed on day 14, day 21 or day 28. Sham-operated rats were fixed on day 28.

### Drug treatment

All drugs were purchased from Sigma-Aldrich. Reserpine and haloperidol were first dissolved in a drop of acetic acid, then diluted in distilled water. SKF-38393 hydrochloride, bromocriptine mesylate and quinpirole hydrochloride were suspended in 0.5% methylcellulose solution. All other drugs were dissolved in physiological saline. Reserpine (1 mg/kg, s.c.), α-methyl-dl-ρ-tyrosine (AMPT) methyl ester hydrochloride (160 mg/kg, i.p.), or p-chloro-dl-phenylalanine (PCPA) methyl ester hydrochloride (320 mg/kg, i.p.) was administered to eight-week-old male BALB/c mice once a day for 3 consecutive days. SKF-38393 hydrochloride (10 mg/kg, i.p.), bromocriptine mesylate (10 mg/kg, i.p.) or quinpirole hydrochloride (1 mg/kg, i.p.) was administered to mice twice a day, 12 h apart, for 3 consecutive days. On the fourth day mice were perfusion-fixed. Haloperidol (10 mg/kg, i.p.) or SCH-23390 hydrochloride (1 mg/kg, i.p.) was administered to mice twice a day for 7 consecutive days.

Eight-week-old D1-null mice, D2-null mice and their wild-type littermates on the C57BL/6J background were treated with reserpine (3 mg/kg, s.c.) once a day for 3 consecutive days. Bromocriptine mesylate (10 mg/kg, i.p.) was administered to nigrostriatal-lesioned rats once a day either for 14 consecutive days from day 14 until day 27, or singly on day 27; rats were perfusion-fixed on day 28. In all experiments, control animals received injections of the diluent vehicle, methylcellulose solution or saline.

### Sample preparation

Rats and mice were perfusion-fixed with a 1∶1 mixture of 4% paraformaldehyde∶HistoChoice (Sigma-Aldrich). Brains were removed and immersed in 4% paraformaldehyde∶HistoChoice for 24 h followed by cryoprotection with 15% sucrose in 0.1 M phosphate buffer for 48 h at 4°C. Cryoprotected brains were frozen using powdered dry ice and sectioned coronally at a thickness of 20 µm on a cryostat for immunofluorescent staining. Needling into contralateral hemispheres was performed before sectioning of the rat brains to identify the laterality of rat brain sections by the presence or absence of holes.

### Immunofluorescent staining

Free-floating brain sections were permeabilized with 0.3% Triton X-100 in PBS with 2% donkey or goat serum, 10 mg/ml bovine serum albumin, and 0.02% sodium azide. The same buffer without Triton X-100 was used for antibody dilution and washes. The following primary antibodies were used: mouse anti-tyrosine hydroxylase (TH, MAB318, Millipore, Billerica, MA, USA, 1∶1000), rabbit anti-AC3 (sc-588, Santa Cruz Biotechnology, Santa Cruz, CA, USA, 1∶500), goat anti-MCHR1 (sc-5534, Santa Cruz Biotechnology, 1∶250), mouse anti-NeuN (MAB377, Millipore, 1∶500), rabbit anti-ADP-ribosylation factor-like protein 13B (Arl13b, a gift from Dr. Tamara Caspary, Emory University, 1∶5000), and mouse anti-S100β (S2532, Sigma-Aldrich, 1∶20000). The primary and secondary antibody incubations were carried out for 16 h at 4°C and for 1 h at room temperature, respectively. The secondary antibodies included Alexa Fluor 594-conjugated goat anti-mouse IgG, Alexa Fluor 488-conjugated goat or donkey anti-rabbit IgG, Alexa Fluor 594-conjugated donkey anti-goat IgG (Invitrogen, Carlsbad, CA, USA, 1∶1000). Nuclei were stained with Hoechst 33342 (Invitrogen).

### Measurement of cilia length, TH intensity and TH-positive cell bodies

Individual neuronal and astrocytic cilia in the dorsolateral striatum were observed by a conventional (nonconfocal) BX50 fluorescence microscope with an UPlanFl oil 100× objective lens (depth of focus = 0.66 µm, Olympus, Tokyo, Japan), and an image of each cilium was captured with a DP50 digital camera (Olympus) if an acceptable amount of sharpness was maintained throughout the total tract of the cilium. The capture of cilia images by microscopic observations and measurements of cilia length using NIH ImageJ software were performed by an investigator who was blinded to treatment of each animal. Intensity of TH immunostaining in each side of the dorsolateral striatum was measured using NIH ImageJ software. Numbers of TH-positive neuronal cell bodies in each side of the substantia nigra pars compacta were counted in three arbitrary midbrain sections per rat.

### Statistical analysis

**Figure 1:**
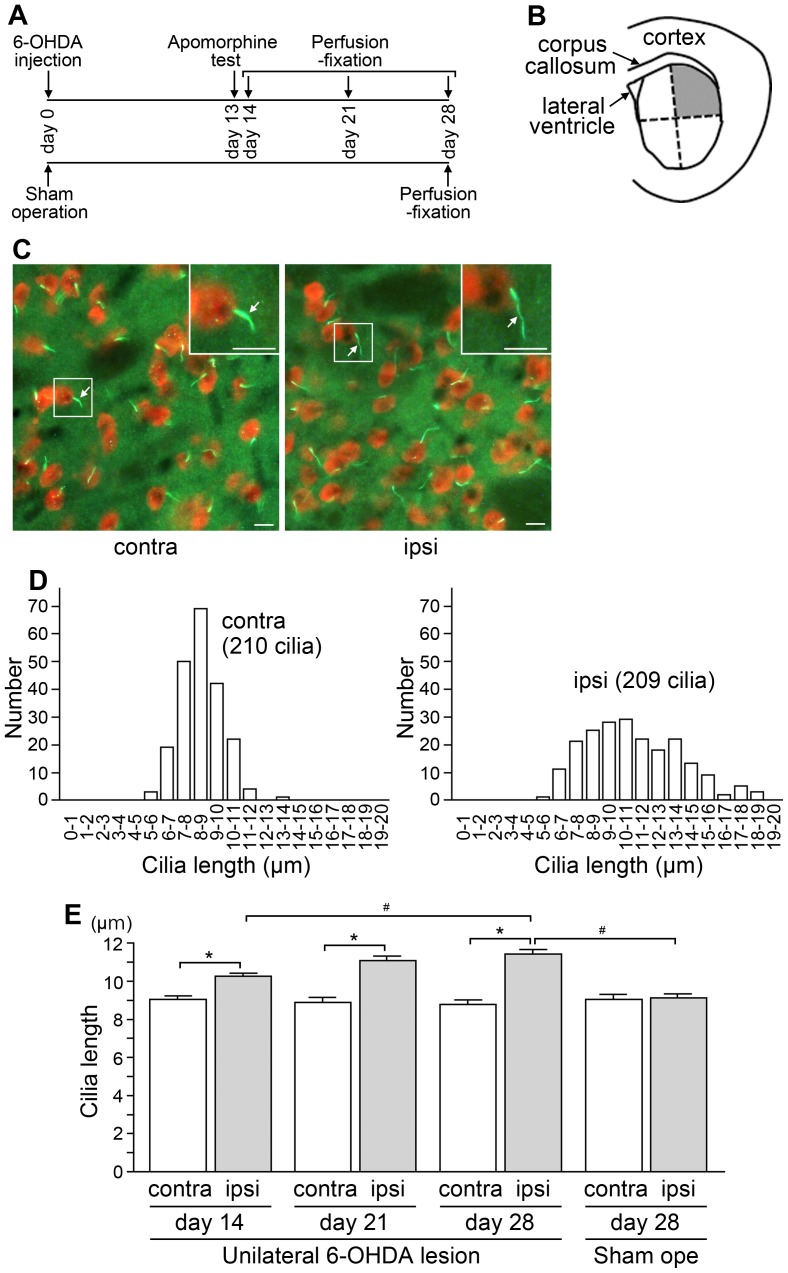
Nigrostriatal dopaminergic lesions lead to the elongation of primary cilia of striatal neurons in rats. (A) Generation and perfusion-fixation of hemi-parkinsonian rats. Rats that received unilateral 6-OHDA injection into the nigrostriatal dopamine pathway on day 0 and displayed asymmetric rotation in the apomorphine test on day 13 were fixed on day 14, 21 or 28. Sham-operated rats were fixed on day 28. Each group included four rats. (B) A drawing indicating the dorsolateral quarter of the striatum (shaded gray), where neuronal cilia were analyzed. (C) An immunofluorescence analysis of the dorsolateral striatum from the unilaterally nigrostriatal-lesioned rat that was fixed on day 28. Primary cilia stained with an antibody to AC3 (green) protruded individually from NeuN-positive neuronal cell bodies (red) on the contralateral (contra) and ipsilateral (ipsi) sides. Insets show higher-magnification views of the boxed areas. Arrows = primary cilia; scale bars = 10 µm. (D) Lengths of AC3-positive cilia protruding from NeuN-labeled neurons were analyzed in the dorsolateral striatum. At least 200 cilia on each side from the respective animals were measured. Histograms show the distributions of cilia lengths on both sides of the striatum from a representative unilaterally-lesioned rat on day 28. (E) The average cilia length in the dorsolateral striatum of both sides from each of four rats in the respective groups was calculated. The data are expressed as means ± SEM of four average values from unilaterally 6-OHDA-lesioned rats fixed on day 14, 21 or 28, and sham-operated (Sham ope) rats fixed on day 28. In the lesioned rats, striatal neuronal cilia were significantly longer on the ipsilateral side than on the contralateral side (*P<0.01). Significant differences were observed between the ipsilateral sides on day 28 of the 6-OHDA-lesioned rats and of the sham-operated (Sham ope) rats, and between the ipsilateral sides on day 14 and on day 28 of the 6-OHDA-lesioned rats (^#^ *P*<0.05).

**Figure 2:**
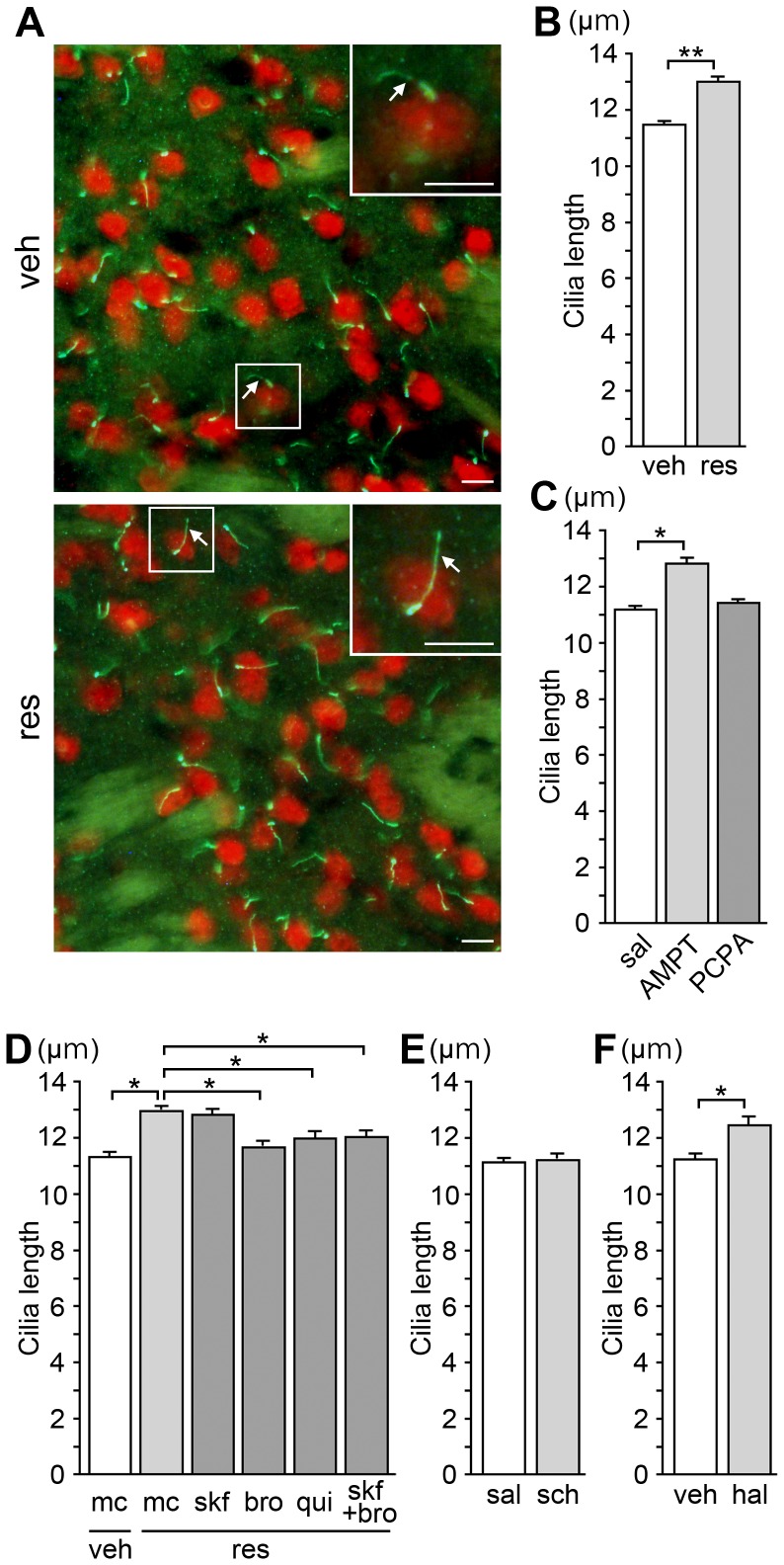
Pharmacological treatments that reduce dopamine inputs cause the elongation of neuronal cilia in the mouse striatum. (A) Immunostaining of AC3 (green) and NeuN (red) in the dorsolateral striatum from adult BALB/c mice treated for 3 days with reserpine (res, 1 mg/kg, s.c.), an alkaloid that depletes monoamine stores, or vehicle (veh). Insets show higher magnification views of the boxed areas. Arrows = primary cilia; scale bars = 10 µm. (B) The average length of at least 200 cilia from each of four mice in the respective groups was calculated; the data are means ± SEM of four average values per group (B–F). The mean lengths of AC3-positive cilia protruding from NeuN-labeled neurons in the dorsolateral striatum were significantly longer in the reserpine-treated mice compared to those in the vehicle-treated mice (**P<0.001). (C) The lengths of striatal neuronal cilia were significantly longer in the mice treated for 3 days with a dopamine synthesis inhibitor AMPT (160 mg/kg, i.p.), but not with a serotonin synthesis inhibitor PCPA (320 mg/kg, i.p.), compared to those in the saline (sal)-treated mice (*P<0.05). (D) Mice were treated for 3 days with the D1 agonist SKF-38393 (skf, 10 mg/kg, i.p., twice a day), or the D2 agonists bromocriptine (bro, 10 mg/kg, i.p., twice a day) or quinpirole (qui, 1 mg/kg, i.p., twice a day) or a suspending agent (methylcellulose solution, mc), in combination with reserpine (1 mg/kg, s.c.). Treatment with bromocriptine, quinpirole or the combination of SKF-38393 and bromocriptine, but not SKF-38393, attenuated the cilium elongation caused by reserpine treatment (*P<0.05). (E, F) The lengths of striatal neuronal cilia were significantly longer in the mice treated for 7 days with the D2 antagonist haloperidol (hal, 10 mg/kg, i.p., twice a day) (F), but not with the D1 antagonist SCH-23390 (sch, 1 mg/kg, i.p., twice a day) (E) compared to those in control mice (*P<0.05).

**Figure 3:**
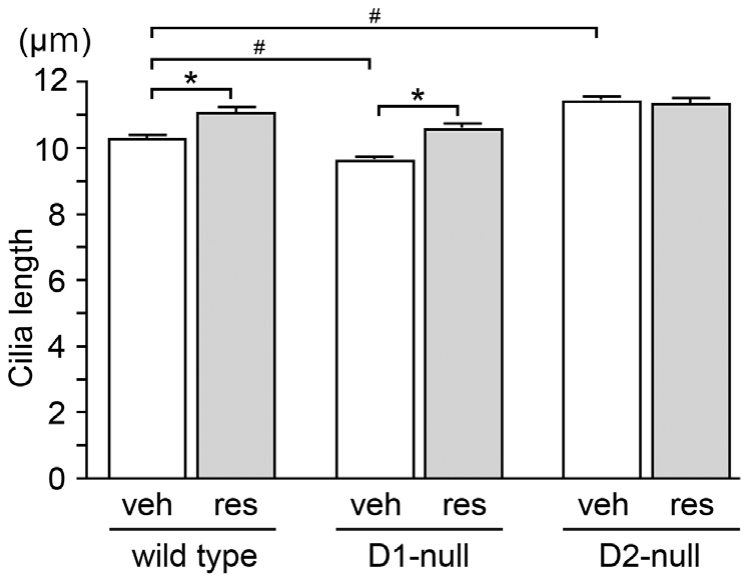
Mice with genetically-inactivated dopamine receptors displayed altered lengths of striatal neuronal cilia. Eight-week-old D1-null mice, D2-null mice and their wild-type littermates on the C57BL/6J background were treated for 3 days with reserpine (3 mg/kg, s.c.) or vehicle. The average length of at least 200 neuronal cilia in the dorsolateral striatum from each of four mice in the respective groups was calculated; the data are means ± SEM of four average values per group (the wild-type groups contained two D1-littermates and two D2-littermates). The vehicle-treated D1-null and D2-null mice revealed significantly shorter and longer neuronal cilia, respectively, compared to the vehicle-treated wild-type controls (^#^ *P*<0.05). Reserpine-treated wild-type and D1-null mice, but not D2-null mice, displayed significantly longer neuronal cilia compared to vehicle-treated animals (*P<0.01).

**Figure 4:**
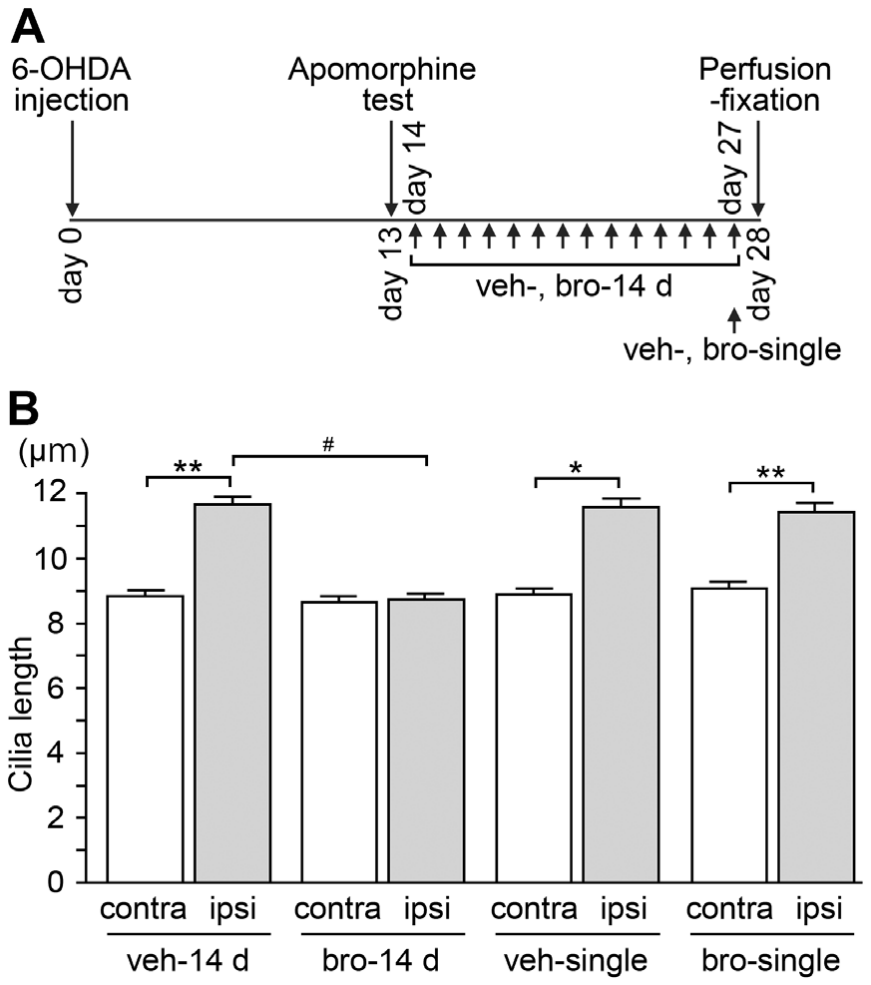
Repeated bromocriptine treatment abrogates the elongation of striatal neuronal cilia in lesioned sides of hemi-parkinsonian rats. (A) Schematic description of bromocriptine and vehicle administration to hemi-parkinsonian rats. 6-OHDA was injected into the unilateral nigrostriatal dopamine pathway on day 0. Bromocriptine or vehicle was administered once a day either for 14 days between the apomorphine-induced rotation test on day 13 and perfusion-fixation on day 28 (bro-14 d, veh-14 d), or singly on day 27 (bro-single, veh-single). (B) The average length of at least 200 neuronal cilia in the dorsolateral striatum of ipsilateral (ipsi) and contralateral (contra) sides from each of four rats in the respective groups was calculated; the data are means ± SEM of four average values per group. Striatal neuronal cilia were significantly longer on the ipsilateral side than on the contralateral side (*P<0.01; **P<0.001) in veh-14 d, veh-single, bro-single groups, whereas the mean cilia length was comparable between the two sides of the striatum in the bro-14 d group. Neuronal cilia on the ipsilateral side were significantly shorter in the bro-14 d group compared to those in the veh-14 d group (^#^ *P*<0.05).

One-way ANOVA with post hoc Dunnett's test was performed for the following comparisons; among data of contralateral sides, among data of ipsilateral sides (Fig. 1E), among data of treatment groups (Fig. 2C,D), among data of vehicle-treated groups, and among data of reserpine-treated groups (Fig. 3). Paired *t*-test was performed for comparisons between data of the contralateral side and data of the ipsilateral side from the respective time points (Fig. 1E, Fig. S1B,D, Fig. S2B) and from the respective treatment groups (Fig. 4B, Fig. S1E,F). Unpaired *t*-test was performed for comparisons in Fig. 2B,E,F, for comparisons between data of the vehicle-treated group and data of the reserpine-treated group from the respective genotypes (Fig. 3), and for comparisons between data of contralateral sides and between data of ipsilateral sides (Fig. S2B). One-way ANOVA with post hoc Dunnett's test was also performed in Fig. 4B and Fig. S1B,D–F for comparisons among data of contralateral sides, and among data of ipsilateral sides. *P*<0.05 was considered significant.

## Results

### Nigrostriatal dopaminergic lesions lead to the elongation of primary cilia of striatal neurons in rats

Rats that received unilateral 6-OHDA injection into the nigrostriatal dopamine pathway at the medial forebrain bundle on day 0 and displayed asymmetric rotation in the apomorphine test on day 13 were fixed on day 14, 21 or 28. Sham-operated rats were fixed on day 28 (Fig. 1A). The immunostaining study revealed that the unilateral 6-OHDA lesion caused the degeneration of both the tyrosine hydroxylase (TH, a dopaminergic neuron marker)-positive neuronal cell bodies in the substantia nigra pars compacta (SNc) (Fig. S1A,B) and the TH-positive nerve endings in the dorsolateral striatum to which SNc dopaminergic neurons project (Fig. S1C,D) in the operated sides on day 14, 21 and 28.

We compared the lengths of AC3-positive cilia protruding from NeuN-labeled neuronal cell bodies on the ipsilateral (operated) and contralateral (not operated) sides of the dorsolateral quarter of the striatum (Fig. 1B–D). In the 6-OHDA-lesioned rats, the neuronal cilia in the striatum were significantly longer on the ipsilateral side than on the contralateral side on days 14, 21 and 28, while the striatal cilia lengths of the ipsilateral and contralateral sides in the sham-operated rats were comparable on day 28 (Fig. 1E). In the 6-OHDA-lesioned rats, the lengths of neuronal cilia on the ipsilateral side displayed a significant increase on day 28 compared to those on day 14 (Fig. 1E). Double immunostaining with anti-MCHR1 and anti-AC3 antibodies revealed the colocalization of these two signaling components to primary cilia on both sides of the dorsolateral striatum of the 6-OHDA-lesioned rats (Fig. S1G).

### Treatment with agents that evoke dopamine deficiency causes elongation of primary cilia of striatal neurons in mice

Since considerable degeneration of the nigrostriatal dopamine pathway in the 6-OHDA-injected side was confirmed by TH immunostaining (Fig. S1A–D), we thought it possible that a loss of dopaminergic release in the striatum led to the observed elongation of primary cilia of striatal neurons (Fig. 1) that express high levels of dopamine receptors [35]. Therefore, we next investigated the effect of dopamine deficiency on the lengths of neuronal cilia in the striatum using mice. We treated adult male BALB/c mice with either reserpine, an indole alkaloid that depletes monoamine stores in nerve terminals, or AMPT, an inhibitor of dopamine synthesis. We measured the lengths of AC3-positive cilia protruding from NeuN-labeled neuronal cell bodies in the dorsolateral striatum in double immunofluorescence-stained sections.

We found that the neuronal primary cilia were significantly longer in the mice treated with either reserpine or AMPT for 3 days compared to those in the control mice, whereas the treatment with PCPA, an inhibitor of serotonin synthesis, had no effect on the cilia length (Fig. 2A–C). Among the five known mammalian dopamine receptor subtypes, type 1 (D1) and type 2 (D2) represent the vast majority of subtypes expressed in the striatum [35]. When bromocriptine or quinpirole, selective D2 agonists, were administered in combination with reserpine for 3 days, the primary cilium elongation was significantly attenuated (Fig. 2D). On the other hand, combined treatment with reserpine and SKF-38393, a selective D1 agonist, failed to attenuate ciliary elongation (Fig. 2D). The co-administration of SKF-38393 and bromocriptine also attenuated the reserpine-induced elongation of neuronal cilia (Fig. 2D).

### Pharmacological blockade of D2 receptor elongates the primary cilia of striatal neurons

We next treated adult BALB/c mice with SCH-23390 or haloperidol, antagonists of D1 and D2 receptors, respectively. The striatal neuronal cilia were significantly longer in the mice treated with haloperidol, but not in those treated with SCH-23390, for 7 days compared to those in the control mice (Fig. 2E,F).

Collectively, the treatment of BALB/c mice with drugs that affect dopaminergic transmission suggests that the diminished dopaminergic inputs elongate the primary cilia of striatal neurons, likely due to downregulation of D2 receptor stimulation.

### Striatal neuronal cilia were elongated and their length was not changed by reserpine treatment in D2-null mice

Next, we examined the length of striatal neuronal cilia in mice with genetically inactivated dopamine receptors. Since 1 mg/kg reserpine treatment for 3 days failed to alter the lengths of striatal neuronal cilia in adult wild-type C57BL/6J mice (data not shown), we administered 3 mg/kg reserpine or vehicle to 8-week-old D1-null mice, D2-null mice and their wild-type littermates on the C57BL/6J background for 3 days. The vehicle-treated D1-null and D2-null mice revealed significantly shorter and longer neuronal cilia, respectively, in the dorsolateral striatum compared to the vehicle-treated wild-type controls (Fig. 3). The reserpine-treated wild-type and D1-null mice, but not D2-null mice, revealed significantly longer neuronal cilia in the striatum compared to the vehicle-treated animals (Fig. 3).

### Repeated treatment with a D2 receptor agonist suppressed the elongation of the primary cilia of striatal neurons in nigrostriatal-lesioned rats

In the unilaterally nigrostriatal-lesioned rats that received bromocriptine (a D2 agonist) for 14 days from day 14 until day 27 (Fig. 4A), there was no significant difference in the neuronal cilia lengths of the dorsolateral striatum between the ipsilateral and the contralateral sides on day 28 (Fig. 4B). Because we observed ipsilateral degeneration of the nigrostriatal dopaminergic neurons after this repeated administration (Fig. S1E,F), we suspected that the suppressed elongation of striatal neuronal cilia in the ipsilateral side was due to the bromocriptine action at the D2 receptor of the striatal neurons. By contrast, the single administration of bromocriptine on day 27 (Fig. 4A) failed to abrogate the elongation of striatal neuronal cilia on the ipsilateral side (Fig. 4B), showing that the bromocriptine-induced suppression of cilia elongation was due not to the acute effect but rather to the chronic effect of the D2 agonist.

### Nigrostriatal dopaminergic lesions did not affect the length of primary cilia of striatal astrocytes in rats

To elucidate whether the elongation of primary cilia following the lack of dopaminergic inputs in the striatum is a neuron-specific phenomenon, primary cilia of astrocytes in the dorsolateral striatum of the unilaterally nigrostriatal-lesioned rats and of the sham-operated rats fixed on day 28 were analyzed. Arl13b, a cilia-localized protein that belongs to the Arf-like small GTPase family [36], is a more suitable marker of primary cilia of astrocytes *in vivo* compared to AC3 (K.M. and K.K., unpublished observation). Double immunostaining with antibodies to S100β, an astrocyte-specific protein [37], and Arl13b revealed that degeneration of the nigrostriatal dopamine projection did not affect the length of astrocytic primary cilia in the striatum (Fig. S2A,B).

## Discussion

The striatum is one of the brain regions where AC3-positive primary cilia are most abundant [5]. Our present findings demonstrated that neuronal cilia in the dorsolateral striatum in rodent brains are relatively long (on average, 9.0 µm in Sprague-Dawley rats, 11.2 µm in BALB/c mice) given the range of mean lengths of neuronal cilia found by Fuchs and Schwark: 2.1 µm to 9.4 µm across the 23 regions of the rat central nervous system [3]. We reported previously that neuronal primary cilia are longer in the striata of mice fed chow containing lithium carbonate, a classical mood stabilizer [25]. The present study is the second to find that neuronal cilia in the striatum were lengthened by pharmacological treatments.

Among the five dopamine receptor subtypes, D1 and D2 are the major receptors expressed in medium spiny neurons [35], which account for 97.7% of the striatal neurons in the rat [38]. D2 receptors are also expressed on striatal interneurons [39], which comprise only 2%–3% of the total neuronal population in the striatum [40] but play key roles in regulating the activities of striatal medium spiny neurons [41]. In the present study, we found that the unilateral destruction of the nigrostriatal dopamine projection elongates the primary cilia of ipsilateral striatal neurons in rats. On day 28, cilia on the ipsilateral side were approximately 1.3-fold longer than those on the contralateral side. This elongation was completely abolished when a D2 agonist, bromocriptine, was administered from day 14 until day 27. Given that the cilia were significantly longer on the ipsilateral side than on the contralateral side on day 14, repeated bromocriptine treatment not only prevented the elongation of the cilia but shortened the cilia to the normal length in the striatum on the ipsilateral side.

**Figure 5:**
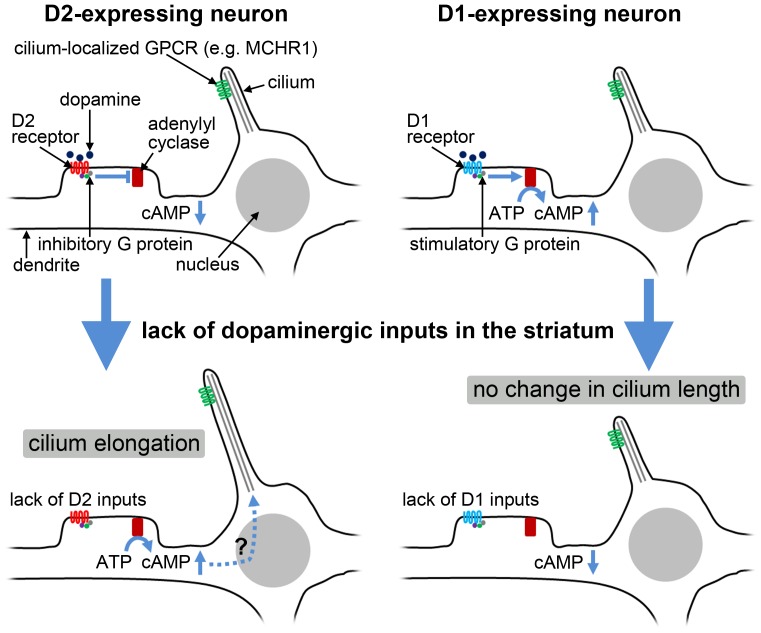
Schematic diagram depicting our findings on the length change of striatal neuronal cilia. It is generally accepted that dopaminergic inputs to D1 and D2 receptors activate or inactivate adenylyl cyclase-mediated generation of cAMP via stimulatory or inhibitory G proteins, respectively. A lack of D1 inputs, which had been expected to decrease the cAMP level, had no effect on the length of neuronal cilia. On the other hand, a lack of D2 inputs, which had been expected to increase the cAMP level, elongated neuronal cilia. Besschetnova et al. demonstrated that cAMP levels positively regulate the length of primary cilia in cultured cells [28], while our results suggest that the alteration of cAMP levels is not likely to fully account for the length change of striatal neuronal cilia.

Mice that received repeated administrations of agents that decrease the striatal dopamine level and of a D2 antagonist displayed significantly longer neuronal cilia in the striatum than the control mice. The combined administration of D2 agonists with reserpine attenuated the elongation of striatal neuronal cilia. Further, D2-null mice displayed significantly longer neuronal cilia in the striatum compared to the wild-type controls. Reserpine treatment failed to alter cilium length in striatal neurons in D2-null mice. These results suggest that elongation of neuronal primary cilia in the dorsolateral striatum following degeneration of the nigrostriatal dopamine projection in rats is attributable to a lack of dopaminergic inputs via D2 receptors. A schematic diagram depicting our findings is presented in Fig. 5. The identification of compounds targeting dopamine receptors as major regulators of flagellar assembly in *Chlamydomonas reinhardtii* [31] may reflect an ancient evolutionary origin of the effects of dopaminergic inputs on the architecture of cilium as well as that of flagellum.

Several studies have demonstrated that dopamine receptors can localize to primary cilia. Mice lacking *Bbs2* or *Bbs4*, two causative genes of BBS, reveal frequent localization of the dopamine D1 receptor at the neuronal cilia of several brain regions [42]. Another study demonstrated that Flag-tagged D1, D2, and D5 subtypes localize to primary cilia of cultured neurons dissociated from the rat striatum [43]. Further, the D2 receptor has been shown to localize to primary cilia of endocrine cells secreting prolactin in the mouse pituitary gland [44]. In our present immunofluorescence analysis, neither D1 nor D2 receptors were detectable at primary cilia in the striata of the ipsilateral and contralateral sides of hemi-parkinsonian rats (data not shown), suggesting that the potential translocation of dopamine receptors into primary cilia is not related to the observed elongation of striatal neuronal cilia, and that a loss of synaptic dopamine transmission underlies the observed cilia elongation.

There are two limitations in the present study. First, given that individual neuronal cilia protruded in nonuniform three-dimensional directions in the striatum (K.M. and K.K., unpublished observation), our data on cilium length could not represent all neuronal cilia in the striatum, owing to the method we used to select neuronal cilia to be measured. As noted in Materials and Methods, an image of each neuronal cilium was captured if an acceptable amount of sharpness was maintained throughout the total tract of the cilium. This means that only cilia extending almost to the coronal plate inside the depth of focus (0.66 µm) were analyzed. Secondly, we were unable to exclude the possibility that the bromocriptine treatment attenuated the elongation of striatal neuronal cilia in a D2-independent manner. Combined treatment with bromocriptine and reserpine attenuated the elongation of neuronal cilia induced by reserpine treatment in BALB/c mice (Fig. 2D) but not in wild-type C57BL/6J mice (data not shown). Consequently, even if this combined treatment does not lead to shorter cilia compared to treatment with reserpine alone in D2-null mice on the C57BL/6J background, we cannot exclude the possibility of a D2-independent effect of bromocriptine treatment.

Further analysis will be required to elucidate how the loss of dopaminergic transmission via D2 receptors leads to the elongation of striatal neuronal cilia. It is generally accepted that cilium and flagellum lengths are regulated by a balance between the assembly and disassembly of axonemal microtubules, which undergo continuous turnover at the distal tip [45]. Thus, either an increase in the assembly rate or a decrease in the disassembly rate will result in equilibration at a longer length. Possible control points for the modulation of cilium length will contain the number of active IFT particles, the velocity of moving IFT particles, and a length-independent disassembly rate [45].

Besschetnova et al. demonstrated that intracellular cAMP levels positively regulate the length of primary cilia through the modulation of protein kinase A activity in cultured renal collecting duct cells and embryonic kidney epithelial cells [28]. Levels of intracellular cAMP generated by adenylyl cyclase, which is activated by D1 inputs and inactivated by D2 inputs, would evoke various cellular events. We consider that alteration of intracellular cAMP levels is not likely to fully account for the observed changes in the length of neuronal cilia in the striatum, for two reasons. First, treatment with a D1 agonist or a D1 antagonist that had been expected to increase or decrease levels of intracellular cAMP, respectively, had no effect on the length of striatal neuronal cilia in BALB/c mice. Secondly, 14-day treatment with the D2 agonist bromocriptine, which had been expected to decrease levels of intracellular cAMP, had no influence on the length of striatal neuronal cilia on the contralateral side of unilaterally nigrostriatal-lesioned rats (Fig. 4B; bromocriptine treatment, 8.67±0.14 µm vs. vehicle treatment, 8.82±0.16 µm (n = 4); *P* = 0.49).

Comparison among vehicle-treated mice with a C57BL/6J genetic background revealed that D1-null mice had significantly shorter neuronal cilia in the striatum compared to wild-type mice, whereas treatment with a D1 antagonist for 7 days failed to alter the striatal neuronal cilia in BALB/c mice. One likely reason for this inconsistency is that the complete loss of D1 inputs throughout development exerted a certain effect on the activity of striatal neurons, which led to a shortening of primary cilia via an unidentified mechanism; namely, that continuous transmission of endogenous dopamine via D1 serves to maintain the proper length of striatal neuronal cilia.

In conclusion, we have demonstrated that a lack of dopaminergic inputs elongates the primary cilia of striatal neurons in rodents, likely due to the absence of dopamine D2 receptor stimulation.

## Supporting Information

Figure S1 **Degeneration of dopaminergic neurons in hemi-parkinsonian rats.** (A, C) 6-OHDA was injected into the unilateral nigrostriatal dopamine pathway on day 0. The upper panels show immunostaining of TH (red), a dopaminergic neuron marker, in the ipsilateral (ipsi) and contralateral (contra) sides of the midbrain (A) and of the dorsolateral striatum (C) on day 28. The lower panels show Hoechst staining (SNc, substantia nigra pars compacta; VTA, ventral tegmental area). Scale bars = 500 µm. (B, D) Significantly lesser numbers of TH-positive neuronal cell bodies in the SNc (B; numbers in three midbrain sections per rat) and lower intensity of TH-immunostaining of nerve endings in the dorsolateral striatum (D) were observed on the ipsilateral side compared to those on the contralateral side on days 14, 21 and 28 of the unilaterally 6-OHDA-lesioned rats (*P<0.001). Significant differences were observed between the ipsilateral sides on day 28 of the 6-OHDA-lesioned rats and of the sham-operated (Sham ope) rats (B, D), and between the ipsilateral sides on day 14 and on day 28 of the 6-OHDA-lesioned rats (B) (^#^ *P*<0.05). (E, F) Significantly lesser numbers of TH-positive neuronal cell bodies in the SNc (E) and lower intensity of TH-immunostaining of nerve endings in the dorsolateral striatum (F) were observed on the ipsilateral side compared to those on the contralateral side in the 6-OHDA-lesioned rats that received bromocriptine or vehicle injection either for 14 days (bro-14 d, veh-14 d), or singly on day 27 (bro-single, veh-single) (*P<0.001). The data are means ± SEM (n = four rats per group (B, D, E, F)). (G) Double staining for AC3 and MCHR1 demonstrates colocalization of these two signaling components to primary cilia on both sides of the dorsolateral striatum of the unilaterally nigrostriatal-lesioned rat on day 28. Arrows = primary cilia; scale bars = 10 µm.
(TIF)

Figure S2 **Nigrostriatal dopaminergic lesions did not affect the length of primary cilia of striatal astrocytes in rats.** (A) An immunofluorescence analysis of astrocytic cilia in the dorsolateral striatum from the unilaterally nigrostriatal-lesioned rat that was fixed on day 28. Primary cilia stained with an antibody to Arl13b (green) protruded from S100β-positive astrocytic cell bodies (red) on the contralateral (contra) and ipsilateral (ipsi) sides. Arrows = primary cilia; scale bars = 10 µm. (B) The average length of at least 200 astrocytic cilia on both sides of the striatum from each of four unilaterally-lesioned rats on day 28 and of four sham-operated (Sham ope) rats on day 28 was calculated. The data are means ± SEM of four average values per group. There was no significant difference in the length of astrocytic cilia between the contralateral and ipsilateral sides of the striatum in the 6-OHDA-lesioned rats and in the sham-operated rats.
(TIF)
