# Supplementary figures and images for: Lack of Dopaminergic Inputs Elongates the Primary Cilia of Striatal Neurons
Source: PLoS One. 2014 May 15;9(5):e97918. doi: 10.1371/journal.pone.0097918 (PMC4022734; doi:10.1371/journal.pone.0097918)

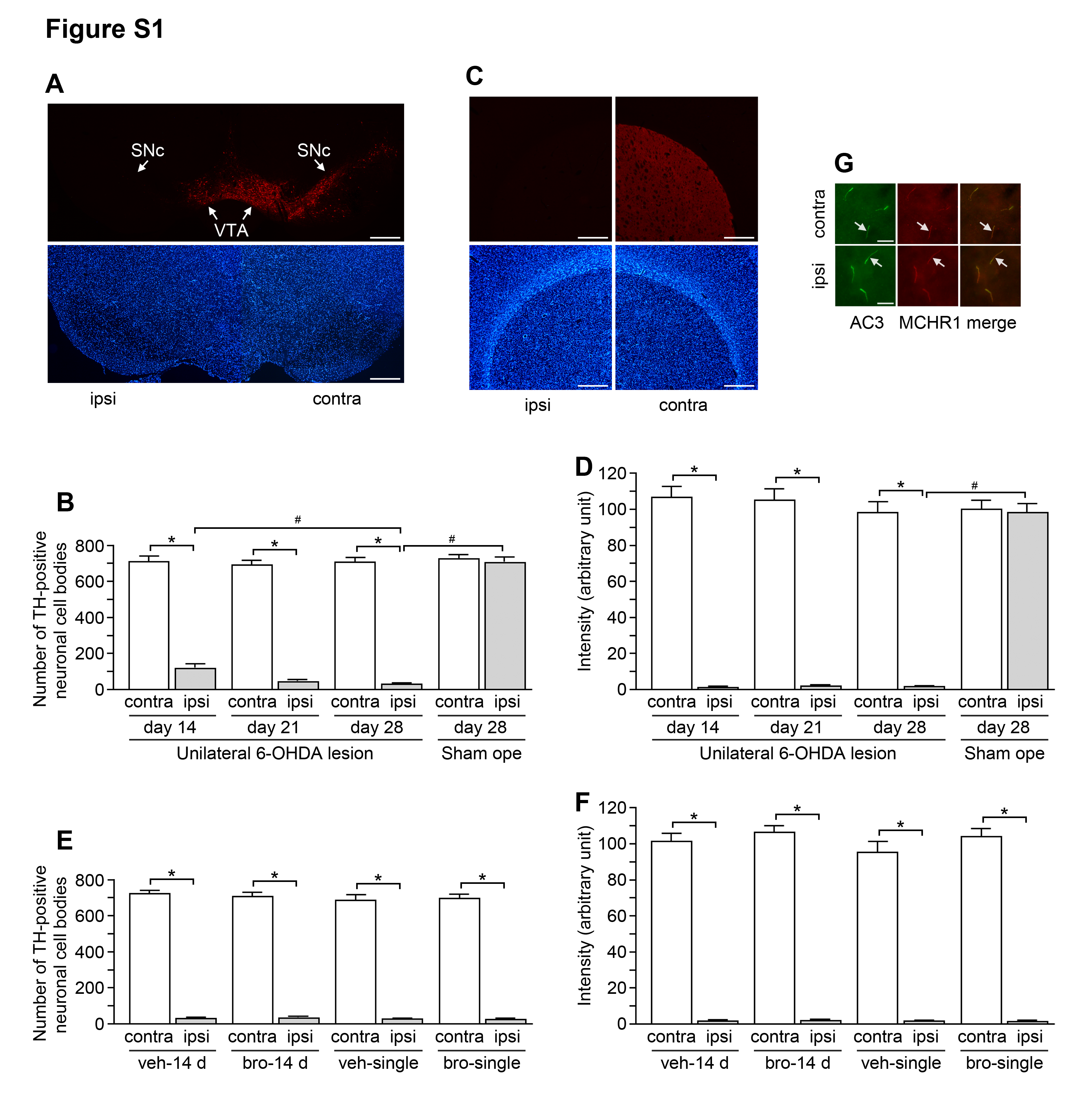

Supplement: Figure S1 — Degeneration of dopaminergic neurons in hemi-parkinsonian rats. (A, C) 6-OHDA was injected into the unilateral nigrostriatal dopamine pathway on day 0. The upper panels show immunostaining of TH (red), a dopaminergic neuron marker, in the ipsilateral (ipsi) and contralateral (contra) sides of the midbrain (A) and of the dorsolateral striatum (C) on day 28. The lower panels show Hoechst staining (SNc, substantia nigra pars compacta; VTA, ventral tegmental area). Scale bars = 500 µm. (B, D) Significantly lesser numbers of TH-positive neuronal cell bodies in the SNc (B; numbers in three midbrain sections per rat) and lower intensity of TH-immunostaining of nerve endings in the dorsolateral striatum (D) were observed on the ipsilateral side compared to those on the contralateral side on days 14, 21 and 28 of the unilaterally 6-OHDA-lesioned rats (*P<0.001). Significant differences were observed between the ipsilateral sides on day 28 of the 6-OHDA-lesioned rats and of the sham-operated (Sham ope) rats (B, D), and between the ipsilateral sides on day 14 and on day 28 of the 6-OHDA-lesioned rats (B) (# P<0.05). (E, F) Significantly lesser numbers of TH-positive neuronal cell bodies in the SNc (E) and lower intensity of TH-immunostaining of nerve endings in the dorsolateral striatum (F) were observed on the ipsilateral side compared to those on the contralateral side in the 6-OHDA-lesioned rats that received bromocriptine or vehicle injection either for 14 days (bro-14 d, veh-14 d), or singly on day 27 (bro-single, veh-single) (*P<0.001). The data are means ± SEM (n = four rats per group (B, D, E, F)). (G) Double staining for AC3 and MCHR1 demonstrates colocalization of these two signaling components to primary cilia on both sides of the dorsolateral striatum of the unilaterally nigrostriatal-lesioned rat on day 28. Arrows = primary cilia; scale bars = 10 µm. (TIF) [file pone.0097918.s001.tif]

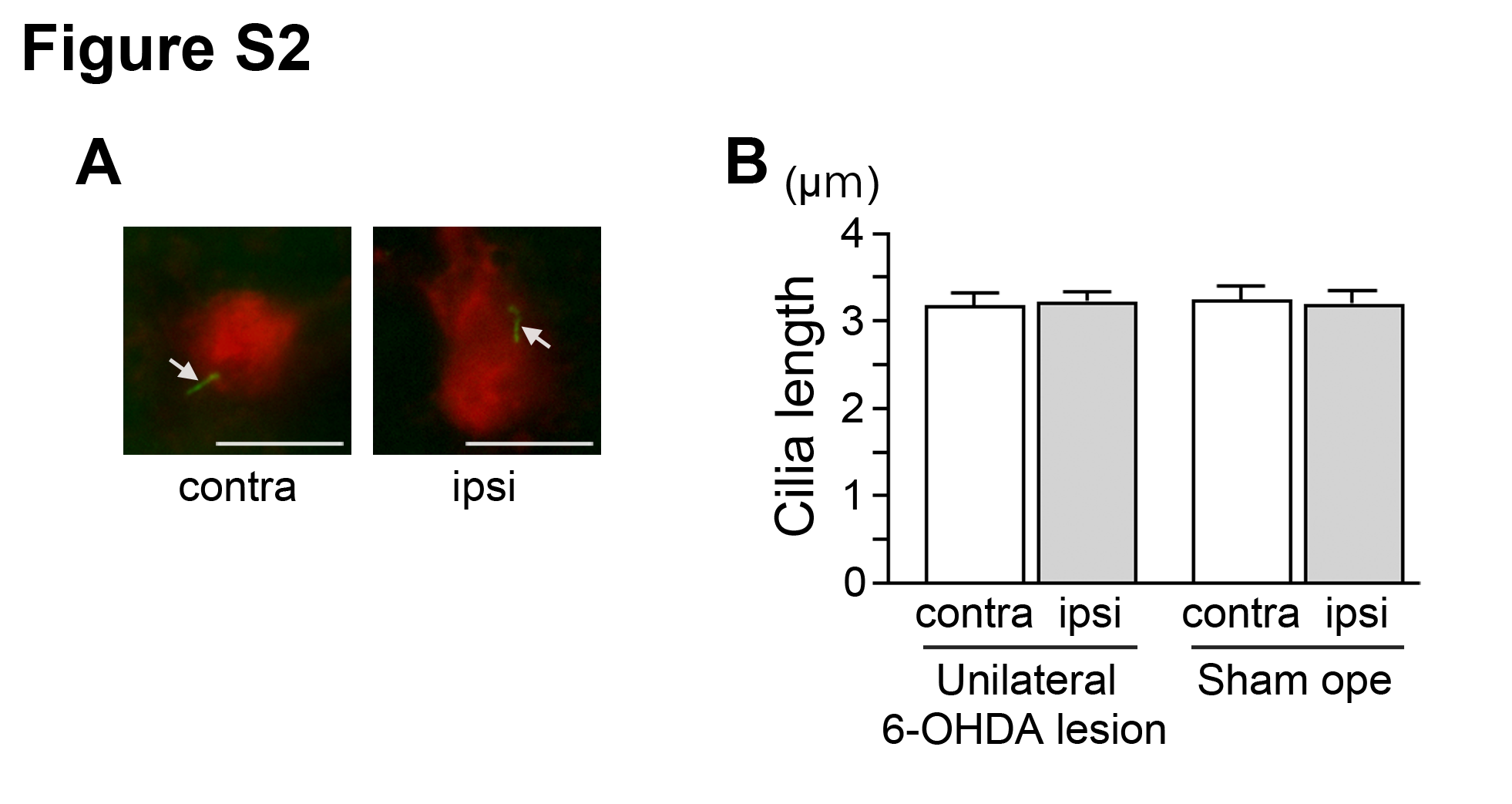

Supplement: Figure S2 — Nigrostriatal dopaminergic lesions did not affect the length of primary cilia of striatal astrocytes in rats. (A) An immunofluorescence analysis of astrocytic cilia in the dorsolateral striatum from the unilaterally nigrostriatal-lesioned rat that was fixed on day 28. Primary cilia stained with an antibody to Arl13b (green) protruded from S100β-positive astrocytic cell bodies (red) on the contralateral (contra) and ipsilateral (ipsi) sides. Arrows = primary cilia; scale bars = 10 µm. (B) The average length of at least 200 astrocytic cilia on both sides of the striatum from each of four unilaterally-lesioned rats on day 28 and of four sham-operated (Sham ope) rats on day 28 was calculated. The data are means ± SEM of four average values per group. There was no significant difference in the length of astrocytic cilia between the contralateral and ipsilateral sides of the striatum in the 6-OHDA-lesioned rats and in the sham-operated rats. (TIF) [file pone.0097918.s002.tif]
